# Molecular analysis of somatic mutations at the *HPRT* locus in lymphocytes of human population exposed to chronic high background natural radiation

**DOI:** 10.1038/s41598-026-43100-y

**Published:** 2026-03-16

**Authors:** Anila Gopinathan, Vinay Jain, Deepak Sharma, P. R. Vivek Kumar

**Affiliations:** 1 Low Level Radiation Research Section, Radiation Biology and Health Sciences Division, Bio-Science Group, Bhabha Atomic Research Centre, Trombay, Mumbai India; 2 Low Level Radiation Research Laboratory, Low Level Radiation Research Section, Radiation Biology and Health Sciences Division, Bio-Science Group, Bhabha Atomic Research Centre, IRE Campus, Kollam, India; 3 Homi Bhabha National Institute (HBNI), Anushaktinagar, Mumbai India

**Keywords:** High level natural radiation areas (HLNRA), Somatic mutant frequency, Chronic low-dose radiation, DNA repair genes, T cell cloning assay, STS marker analysis, Gene expression, Radiation-induced mutagenesis, Biomarkers, Diseases, Genetics, Molecular biology

## Abstract

Populations residing in high-level natural radiation areas (HLNRA) along the monazite-rich coastal belt of Kerala, India, are chronically exposed to low-dose ionizing radiation, with lifetime cumulative doses exceeding 1000 mGy. Although the biological effects of acute high-dose exposures are well established, the long-term health consequences of chronic low-dose or low-dose-rate ionizing radiation (LDIR) remain poorly understood. To address this knowledge gap, we examined the frequency and molecular spectrum of somatic mutations at the hypoxanthine–guanine phosphoribosyltransferase (*HPRT*) locus-a sensitive biomarker of mutagenesis-in peripheral blood lymphocytes from 37 healthy adults residing in normal-level (NLNRA, N = 12) and high-level (HLNRA, N = 25) natural radiation areas. Using a T-lymphocyte cloning assay, PCR-based molecular profiling, and sequence-tagged site (STS) analysis, we found no significant difference in *HPRT* mutant frequency (*p* = 0.43), mutation spectrum (*p* = 0.52), or deletion subtype distribution (*p* = 0.69) between HLNRA and NLNRA groups. The extent of genomic deletions, spanning 0.09–1.19 Mb, was also comparable between HLNRA and NLNRA populations. However, gene expression profiling revealed significant alterations in DNA repair genes, including *KU80*, *DDB2*, *MSH6*, *PCNA*, and *RAD50*, suggesting that *HPRT* mutation may influence DNA damage response pathways. Our findings indicate that chronic exposure to natural background radiation does not elevate somatic mutation frequency or induce large-scale genomic alterations but may modulate DNA damage response and repair gene regulation. This study provides critical human data for refining low-dose radiation risk models and understanding mechanisms of genome stability and adaptation in chronically exposed populations.

**Supplementary Information:**

The online version contains supplementary material available at 10.1038/s41598-026-43100-y.

## Introduction

Natural radiation is ubiquitous across the Earth; however, its intensity exhibits significant geographical variability, leading to specific locations characterized by elevated levels of natural radioactivity. On a global scale, regions designated as High Level Natural Radiation Areas (HLNRAs) are primarily located in India, Iran, Brazil, and China^1^. In India, the southwestern coastal expanse, measuring approximately 59 kms in length and 0.5 kms in width along the Arabian Sea in Kerala, stretches from Sakthikulangara (8.9230° N, 76.5527° E) in the Kollam district to Purakkad (9.3535° N, 76.3653° E) in the Alappuzha district. This particular area is distinguished by its monazite deposits, which constitute approximately 1% of the beach sand and contain thorium-232 (8–10%), uranium-238 (0.3%), along with their corresponding decay products, thus defining it as a significant HLNRA. The uneven distribution of monazite along the beach results in significant variations in background radiation dose levels, ranging from < 1.0 mGy/year to > 45.0 mGy/year. The local human population has been residing here for generations. The southwest coastal region of Kerala stands out among the world’s HLNRAs due to its high population density, diverse dose levels, presence of built-in controls, and a stable, non-migratory community^2^. Since ionizing radiation (IR) is recognized as a DNA-damaging agent, Kerala’s HLNRAs provide a significant opportunity for research on the effects of chronic low-level radiation on human health.

IR induces damage by depositing energy within tissues, leading to the ionization of atoms and molecules. This process results in direct damage to DNA, manifested as single-strand and double-strand breaks, and indirect damage by creating reactive oxygen species (ROS), adversely impacting DNA, proteins, and cellular membranes. The cellular consequences include apoptosis, necrosis, or mutations, which may ultimately contribute to carcinogenesis^3,4^. The genetic alterations associated with high-dose ionizing radiation (HDIR) are well documented, however, the effects of low-dose ionizing radiation (LDIR), particularly doses below 100 mSv, remain uncertain^5,6^. LDIR may have beneficial effects, known as radiation hormesis, where small exposures can stimulate DNA repair mechanisms, enhance immune responses, reduce oxidative stress, and improve cellular function. Therefore, studying populations that have lived in regions with high natural radiation levels for generations provides a unique chance to investigate potential genetic changes in these individuals resulting from chronic low-dose radiation exposure.

In the past, several studies have been conducted involving both newborns and adults within the southwest coastal Kerala population to investigate the biological and health impacts of chronic low-dose natural radiation exposure. No significant differences were observed between newborns from HLNRA and NLNRA regarding the incidence of major congenital anomalies, chromosomal aberrations, and the spontaneous frequency of micronuclei^7–9^. Furthermore, studies conducted on the adult population concerning structural chromosomal alterations, micronuclei frequency, telomere length, mental retardation, cleft lip/palate, and the prevalence of cancer did not indicate any significant difference between HLNRA and NLNRA^10–14^. Nevertheless, age-related response to spontaneous DNA damage was influenced by the area of residence of the individuals (HLNRA/NLNRA) when assessed for DNA strand breaks using the alkaline comet assay and DNA double strand breaks using $\gamma$ H2AX foci^15,16^. In addition, some of the studies suggest adaptive responses that may help mitigate radiation-induced damage^17–20^. Therefore, investigating somatic mutations in regions characterized by elevated background radiation levels is essential for understanding the potential genetic effects of chronic low-dose radiation exposure. Somatic mutations, which occur in non-reproductive cells, may play a significant role in the onset of cancer and other diseases in humans. Therefore, by analyzing mutation rates and molecular patterns within this population, it is possible to determine whether chronic radiation exposure is associated with an increased mutation rate, thereby offering valuable insights into human responses to environmental radiation exposure. Furthermore, these studies contribute to assessing the validity of the linear no-threshold model (LNT) for radiation risk and aid in the refinement of radiation protection guidelines.

We have analysed the molecular spectrum of somatic mutations at the *HPRT* locus, a well-characterized genetic marker located on the X chromosome. The *HPRT* gene encodes an enzyme that is involved in the purine salvage pathway, which recycles purines to synthesize nucleotides essential for the formation of DNA and RNA^21^. Several advantages are associated with this gene, including its location on the X chromosome, the fact that a single mutational event in adult males results in a mutant phenotype, the gene’s size that provides a relatively large target for mutation, and the non-essential nature of the gene, which does not exclude the mutants from the body’s system^22,23^. The T lymphocyte cloning assay has been widely and successfully employed to evaluate *HPRT* mutant frequency in human lymphocytes, providing a reliable measure of genomic instability caused by radiation or chemical exposures^24,25^. Several studies have used this assay to assess *HPRT* mutant frequency in healthy subjects and individuals who have been directly or indirectly exposed to ionizing radiation, including radiotherapy patients and atomic bomb survivors^26–34^. The basic principle of the assay is that, in the presence of a toxic purine analogue such as 6-thioguanine (6TG) in the culture medium, *HPRT* mutant cells, which cannot phosphoribosylate 6TG, survive by utilizing the de novo pathway. In contrast, normal cells phosphoribosylate and activate the 6TG into a toxic form that gets incorporated into their DNA, resulting in cell death. Studying the molecular spectrum of *HPRT* mutations is highly important because it reveals specific mutational signatures of environmental exposures and acts as a sensitive biomarker for assessing genomic instability and health risks^35–38^. It therefore helps determine whether chronic exposure to low-dose radiation causes any unique mutations or the mutational spectrum resembles to the individuals from areas with normal natural radiation levels.

Our study focused on determining somatic mutation rate in response to chronic radiation and molecular characterization of deletion mutations at the *HPRT* locus. These mutations reflect genomic instability and DNA damage, particularly in the context of environmental factors such as radiation exposure. Analyzing deletion patterns enables the identification of specific mutations caused by radiation exposure, thereby offering valuable insights into DNA repair mechanisms and cancer development. Consequently, this study is highly relevant as it addresses the current knowledge gap in understanding the nature and frequency of mutations in response to chronic low-dose radiation exposure in the human population. We also characterized the extent of large deletions beyond the *HPRT* locus by using PCR primers targeting 11 X-linked Sequence-Tagged site (STS) markers, covering an estimated area of 3.3 Mb. Additionally, the potential role of the *HPRT* gene in maintaining genomic stability was examined by analyzing the transcriptional profile of DNA damage response (DDR) and repair genes in *HPRT* mutants.

## Materials and methods

### Study group and blood sample collection

The study involved 37 healthy adult males between the ages of 30 and 65 years. The study participants shared similar socio-economic backgrounds, lifestyles, and dietary patterns. A questionnaire was used to gather information on participants’ age, occupation, personal habits such as smoking and alcohol use, as well as medical radiation exposure. Among the lifestyle factors, smoking and alcohol consumption were included in the analyses. The participants were categorized into three groups according to their annual absorbed dose: Group I [NLNRA ≤ 1.50 mGy/year, N = 12], Group II [HLNRA, Low Dose Group (LDG) 1.51–10.00 mGy/year, N = 12] and Group III [HLNRA High Dose Group (HDG) 10.01–35.00 mGy/year, N = 13]. Migration into and out of the study area was minimal^39^, and all participants were lifelong residents of their respective radiation zones.

**Fig. 1:**
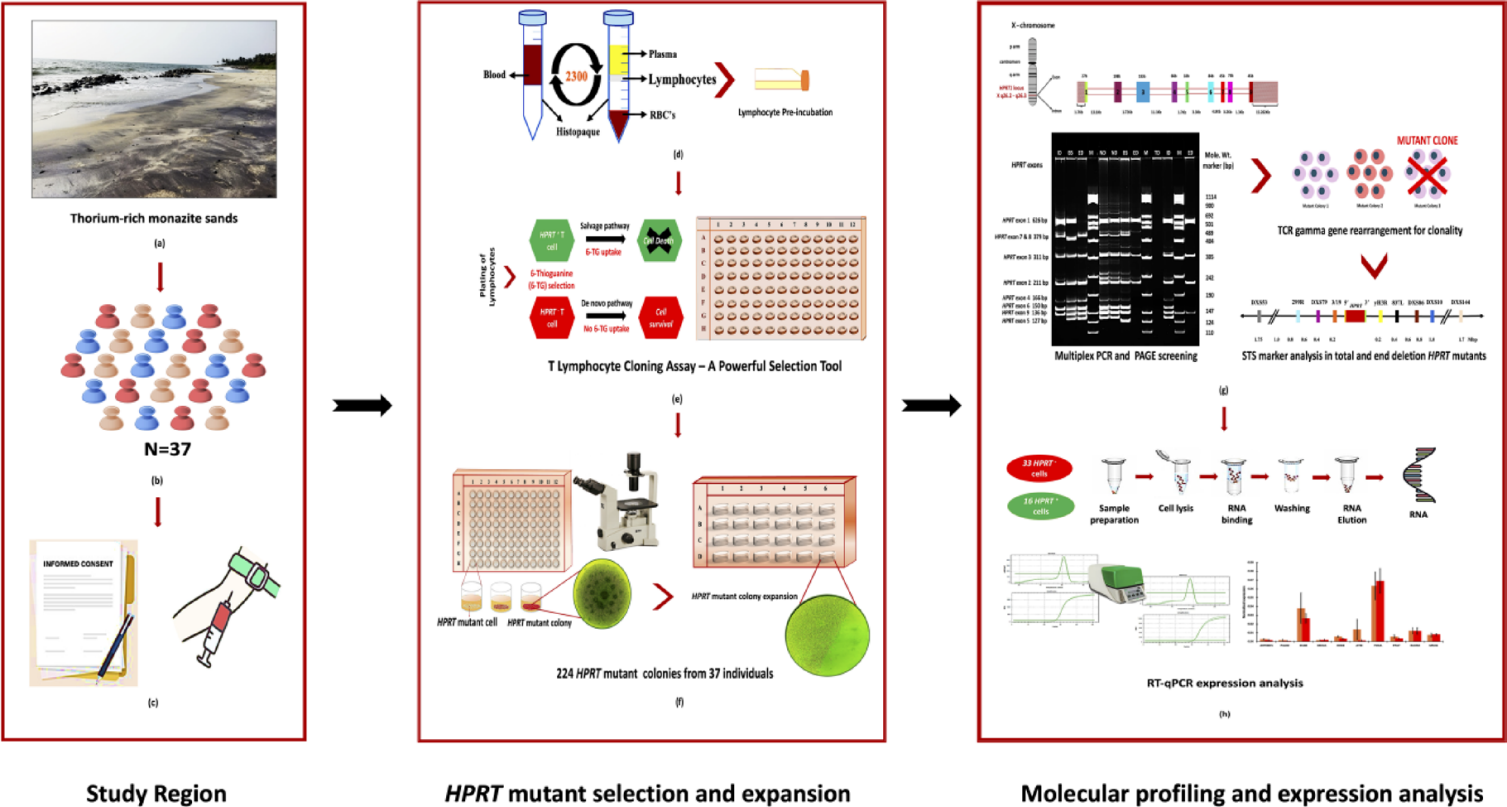
Workflow outline of the study (**a**) Representative photograph of thorium-rich monazite beach sand from the study region. (**b**) Sample tracing and identification. (**c**) Securing informed consent and collection of blood samples. (**d**) Isolation of peripheral blood lymphocytes and pre-incubation. (**e**) T-lymphocyte cloning assay: plating lymphocytes in 96-well plates with 6-thioguanine (6-TG) selection and incubation at 37 °C for 14 days. (**f**) Screening of 96-well plates using phase-contrast microscopy, transfer of positive wells to 24-well plates, and expansion of mutant colonies by sub-culturing. (**g**) Molecular characterization of *HPRT* mutants: deletion spectrum analysis by multiplex PCR and PAGE, T-cell receptor gamma gene rearrangement analysis for in vivo clonality assessment, and mapping of the *HPRT* locus in total and end-deletion mutants. (**h**) Gene expression analysis of *HPRT* mutants using RT-qPCR and downstream data analysis.

Approximately 10 ml of whole blood was drawn from each participant via venipuncture using sterile heparin-coated vacutainer tubes (BD Vacutainer). All blood samples were collected with written informed consent approved by the Medical Ethics Committee (Project no. BHMECNP/09/2023), Bhabha Atomic Research Centre, Trombay, Mumbai, India, and all the experiments were performed in accordance with relevant guidelines and regulations. Following blood collection, the samples were promptly transported to the laboratory in refrigerated conditions for further processing. A schematic overview of the study design is provided in Fig. 1.

### Dosimetry

The external gamma radiation levels were measured outside and inside each residence using a halogen-quenched Geiger-Muller (GM) survey meter (Type ER-709, Nucleonix Systems, India). Measurements were taken at a height of one meter both indoors (specifically in the room most frequently occupied) and outdoors (near the main entrance) on the same day of blood sample collection. For each location, three readings were recorded and averaged to obtain a representative exposure value. The recorded exposure rates, expressed in micro-roentgen per hour (µR/h), reflected ambient gamma radiation in air and were converted to annual absorbed dose (mGy/year) using the standard conversion factor of 0.0765 [= 0.873 × 24 h × 365 days × 10^−5^]. In the present study, dose estimates were based on external gamma radiation measurements and internal exposures from inhalation and ingestion were not included. Individual gamma dose was calculated using the formula: Annual dose = 0.5 (occupancy factor) × annual indoor dose + 0.5 × annual outdoor dose, where the occupancy factor (OF) of 0.5 accounts for time spent indoors and outdoors, based on sex- and age-specific lifestyle data by Nair et al.^40^.

### Peripheral blood mononuclear cells (PBMCs) separation and pre-incubation

PBMCs were isolated by layering an equal volume of whole blood over the Histopaque-1077 solution (Sigma-Aldrich, 1077-1), followed by centrifugation (Eppendorf Centrifuge 5804R) at 400 g for 30 min. Subsequently, the opaque interface containing mononuclear cells was transferred to a sterile 15 ml centrifuge tube, and the cells were washed with RPMI-1640 medium (Himedia, AL028) at 250 g for 10 min each. PBMCs were preincubated in a 25 cm^2^ tissue culture flask (Tarsons 950040) for 22 to 24 h at a concentration of 10^6^ cells/ml in cell culture medium (CCM) containing RPMI-1640 medium (HEPES modification, Himedia, AL060), enriched with 20% AIM-V serum-free medium (Invitrogen), 15% fetal bovine serum (Himedia, RM10915), 1% antibiotics (100Uml^−1^ penicillin, 100µgml^−1^ streptomycin, Sigma-Aldrich, P0781), 50 µM 2-mercaptoethanol (Sigma-Aldrich, M3148), 1 mM sodium pyruvate (Sigma-Aldrich, S8636), and 2 mM L-glutamine (Sigma-Aldrich, G7513) to remove monocytes adhering to the bottom of the culture flask.

### T lymphocyte cloning assay

**Fig. 2:**
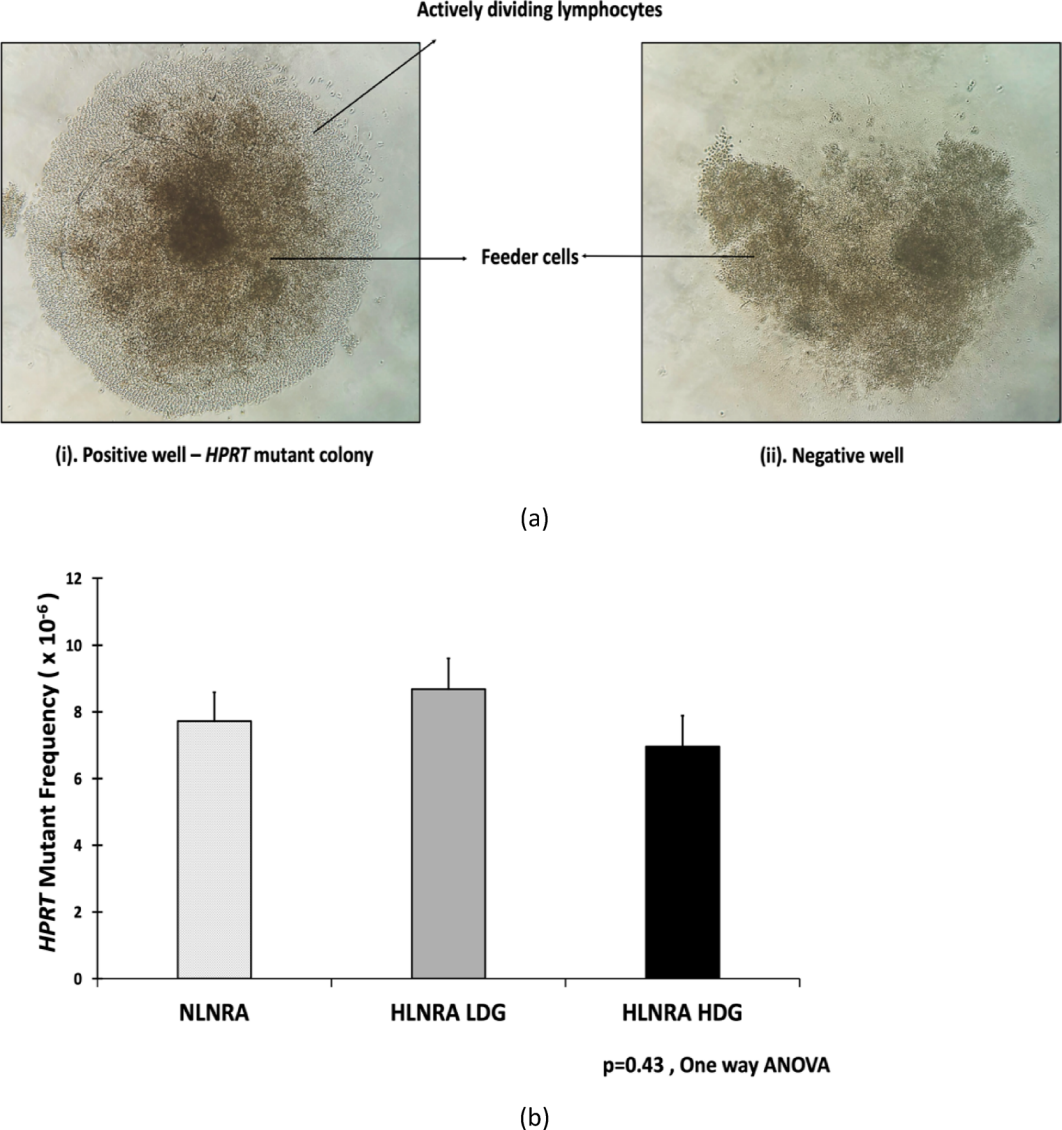
(**A**) Phase contrast microscopy images of a positive well with *HPRT* mutant T lymphocyte colony (i), developed over a 14-day incubation period at 37℃ in the presence of the selection agent 6TG. The central dark core represents the feeder cells, while the lighter periphery signifies the actively dividing *HPRT* mutant T lymphocytes. The absence of a colony is illustrated in (ii), depicting a negative well. (**B**) The mean somatic mutant frequencies at the *HPRT* locus in NLNRA, HLNRA LDG, and HLNRA HDG.

The assay was conducted according to the standardized protocol outlined by Hou et al. in 1999, with minor modifications. After preincubation, lymphocytes were transferred into 96-well round-bottom microtiter plates (Genetix Biotech Asia, 34096) using fresh cell culture medium (CCM) as mentioned above. The CCM was supplemented with phytohemagglutinin (PHA-P) (Sigma-Aldrich, L1668) at 5 µg/ml and human recombinant Interleukin-2 (IL-2, Prospec, CYT-209-b) at 400 U/ml. Lymphocytes were plated at a density of 2 cells per well in non-selected plates (2 plates per sample), and 2 × 10^4^ cells per well in 6TG-selected plates (5 plates per sample). All wells also received 90 Gy irradiated human lymphoblastoid TK6 _LLrrL_ feeder cells^41^ at 1 × 10^4^ cells/well. In selected plates, 6-thioguanine (6TG, Sigma-Aldrich, A4660), was added at a final concentration of 2.5 µg/ml. Both sets were incubated for 14 days at 37 °C in a humidified environment with 5% CO_2_. These plates were scored for lymphocyte colonies as shown in Fig. 2a., using an inverted phase contrast microscope (Axiovert, Carl-Zeiss GmbH).

Cloning efficiencies (CE) of lymphocytes cultured under both non-selected and 6-TG selected conditions were determined by calculating the proportion of negative wells, assuming a Poisson distribution. CE is given by the formula CE = **–** ln P_0_/N, where P_0_ is the fraction of wells without colonies, and N is the number of cells per well. Mutant frequency (MF) was then computed as the ratio of CE in 6-TG selected plates to that in non-selected plates.

$$MF = \frac{Cloning\;efficiency\;of\;the\;6 - thioguanine\;selected\;plate\;(CEs)}{{Cloning\;efficiency\;of\;the\;non - selected\;plate(CE_{{{\mathrm{NS}}}} )}}$$

### Mutant colony expansion, DNA isolation and quantification

*HPRT* mutant lymphocyte colonies obtained from 6-TG-selected plates were transferred to a 24-well plate, containing feeder cells to facilitate continued growth. The colonies were expanded through subsequent subculturing to enhance the cell population. Upon reaching an adequate number of cells (10–15 × 10^6^ cells), genomic DNA was isolated from each mutant colony ($\sim$ 2–2.5 × 10^6^ cells) utilizing the Wizard® Genomic DNA Purification Kit (Promega, A1120). The concentration and purity of the DNA are evaluated using a Nanodrop spectrophotometer (MaestroGen), employing standard A260/280 and A260/230 ratios.

###  Molecular analysis of *HPRT* mutant colonies using multiplex polymerase chain reaction (m-PCR)

Multiplex-PCR (m-PCR) was performed on genomic DNA isolated from mutant colonies to amplify exons 1–9 of the *HPRT* gene. The primers used in the PCR reaction were adapted from the work of Gibbs et al.^42^, O’Neill et al.^43^ and Park et al. ^44^ (Supplementary Table 1). Exons 2–9 were amplified in a single multiplex reaction; however, exon 1 was amplified separately due to its large size of the amplified product. The simultaneous amplification of the eight *HPRT* exons (2–9) was accomplished using seven pairs of oligonucleotide primers (with exons 7 and 8 amplified in one fragment) in a single PCR run. In the multiplex reaction, 250 ng of DNA was combined with 14 primers (50 pmol each) in a total volume of 50 µl, which contained 70 mM KCl, 14 mM Tris HCl, 2.5 mM MgCl_2_, and 500 µM of deoxyribonucleotide triphosphate (dNTP) mix (Promega, U1515), along with 2.5 units of AmpliTaq DNA polymerase (Applied Biosystems, N8080161). Amplification was conducted using a preheating at : 94 °C for 5 min; followed by 25 cycles of 94 °C for 50 s, 52 °C for 45 s, and 68 °C for 1 min, with a final extension at 68 °C for 1 min. Exon 1 was amplified in a separate reaction using 250 ng of DNA template and 50 pmol of primer in a total volume of 50 µl, which included 63 mM Tris HCl, 16.8 mM (NH_4_)_2_SO_4_, 6.7 mM MgCl_2_, 1.5 mM dNTP mix, 2.5 units of Taq DNA polymerase (Roche), 10% DMSO, 5 mM β-mercaptoethanol, and 6.8 µM EDTA. The reaction parameters were set as follows: 94 °C for 4.5 min; 25 cycles of 94 °C for 30 s, 65 °C for 30 s, and 68 °C for 1.4 min, followed by a final extension at 68 °C for 7 min. Genomic DNA from PBMCs was used as positive control for *HPRT* exon amplification. DNA from the TK6_LLrrL_ cell line with *HPRT* total deletion and no DNA template reactions served as negative controls. All PCR procedures were performed in a Mastercycler Nexus (Eppendorf). PCR products were then analysed using 8% polyacrylamide gel electrophoresis (PAGE) and visualized with SYBR Green I to detect deletions in the *HPRT* gene among the various mutants.

### Validation of Mutants using T-cell receptor rearrangement pattern analysis

Clonality of in vivo *HPRT* mutant T-cell populations was assessed by analyzing T-cell receptor (TCR) γ gene rearrangements using multiplex PCR (m-PCR), as described by Ma et al.^45^. The TCR γ gene is composed of variable (V), joining (J), and constant (C) gene segments, each comprising multiple subgroups. Amplification occurs only when V and J segments are rearranged, as the primers are positioned far apart in the germline configuration, preventing amplification in non-rearranged (germline) DNA. For the m-PCR, oligonucleotide primers specific to the V gene segments were used at the 5′ end, while J gene segment-specific primers were used at the 3′ end. Each mutant T-cell clone was analysed using three separate PCR reactions, each containing one of the J segment primers in combination with all three V segment primers. The resulting amplicons included the V gene segment, the V–J recombination junction, and the J gene segment, confirming TCR γ gene rearrangement and enabling detection of clonal expansion. PCR products were analysed by electrophoresis on 2% agarose gels stained with ethidium bromide. Primer sequences and thermocycling conditions are provided in Supplementary Tables 2 and 3.

### Sequence tagged sites (STS) analysis of Total and End deletion *HPRT* mutants

The extent of deletion in adjoining regions of *HPRT* gene locus were mapped utilizing 11 proximal and distal sequence-tagged site (STS loci) markers closely linked to the *HPRT* locus. As illustrated in Supplementary Fig. 1, proximal or centromeric STS markers were located at the 5ʹ end and distal or telomeric markers were positioned at the 3ʹ end within the Xq26 region surrounding the *HPRT* locus^46–51^. Only total deletion (TD) and end deletion (ED, both 5′ and 3′) *HPRT* mutants were analysed to detect deletions beyond *HPRT* gene locus. The sequences of the PCR primers and amplification conditions for these markers are detailed in Supplementary Table 4. Each STS marker was amplified individually, and the resulting PCR products were analysed through agarose gel electrophoresis.

### Gene expression of DNA repair genes: RNA isolation from *HPRT* mutant and wild-type lymphocyte colonies

Total RNA was isolated from the 33 *HPRT* mutant and 13 *HPRT* wild-type lymphocyte colonies using the QIAGEN RNeasy Micro Kit (74034) as per manufacturer’s instructions. In brief, around 2–2.5 × 10^6^ mutant and wild-type colonies were prepared for RNA isolation. The cells were lysed in the provided RLT buffer containing $\beta$-mercaptoethanol. The lysate was then homogenized, and ethanol (70%) was added to facilitate RNA binding to the column. Following several wash steps with wash buffers (RW1 and RPE) and ethanol (80%), total RNA was eluted in RNase-free water. The concentration and purity of the RNA were evaluated with a Nanodrop spectrophotometer (MaestroGen) using standard A260/280 and A260/230 ratios.

### Preparation of cDNA and real-time quantitative PCR (RT-qPCR)

For each aliquot of samples, total RNA (400 ng) was reverse transcribed to cDNA using the transcriptor high-fidelity cDNA synthesis kit (Roche 05081963001) as per manual instructions. Random hexamer primer at a final concentration of 60 $\mu$M was used for reverse transcription. cDNA was used to study the mRNA levels of selected DNA damage response and repair genes (*ATM, ARTEMIS, PALB2, BRCA1, DDB2, KU80, PCNA, PPAT, RAD50* and *MSH6*). RT-qPCR was performed on 96-multiwell plates using SYBR Green chemistry in CFX Opus 96 Real-Time PCR System (BIORAD). All reactions were conducted in triplicate and *GAPDH* was used as endogenous control for all the genes. All the primers were obtained from Integrated DNA Technologies (IDT) and optimized using different parameters. Only the primers giving clean amplification curves with single specific amplicon were used in the study. The primer sequences are listed in Supplementary Table 5. Each RT-qPCR reaction was carried out in a total volume of 10 $\mu$l, which included 1X FastStart SYBR Green master mix, 10 pmols of both forward and reverse primers, and a diluted cDNA template. A total of 50 cycles of real-time qPCR were performed for all the genes. The thermocycling conditions were as following, a pre-incubation at 95 °C for 5 min, denaturation at 95 °C for 10 s, annealing at 58 °C for 30 s, and extension at 72 °C for 30 s. The melting curve analysis involved three stages: melting at 95 °C for 1 min, an annealing step at 58 °C for 1 min, extension at 72 °C, and a final step at 40 °C for 10 s. The representative amplification and melting curves are shown in Supplementary Fig. 2.

### Statistical analysis

One-way ANOVA was used to compare non-selected and 6-TG-selected cloning efficiencies, as well as *HPRT* mutant frequencies across the study groups (NLNRA, HLNRA LDG, and HLNRA HDG). *HPRT* mutant frequency data from all subjects were also analysed using a general linear model (GLM), which included residential area (NLNRA = 0, HLNRA = 1), smoking status (non-smoker = 0, smoker = 1), and alcohol consumption (non-alcoholic = 0, alcoholic = 1) of individuals as indicator covariates*.* Chi-square analysis was employed to compare the frequency of different deletion mutations between study groups. Gene expression profiles of DDR and repair genes between *HPRT* mutant and wild-type lymphocyte colonies were compared using Student’s t-test. All analyses were carried out in SPSS version 10.0, and a *p*-value $<$ 0.05 was considered statistically significant.

## Results

The study aimed to determine the frequency and molecular spectrum of somatic mutations at the *HPRT* locus in 37 individuals from normal (NLNRA) and high-level natural radiation areas (HLNRA) in Kerala. The NLNRA group comprised 12 individuals (mean age: 44.83 ± 9.56 years), while the HLNRA group included 25 individuals (mean age: 49.56 ± 8.68 years). There was no statistically significant difference in mean age between the two groups (*p* = 0.16; Student’s t-test). The mean annual absorbed dose was 1.23 ± 0.17 mGy/year in NLNRA group and 11.62 ± 9.88 mGy/year in HLNRA group. Within the HLNRA group, individuals were further categorized into low-dose group (LDG) and high-dose group (HDG), with mean doses of 3.20 ± 1.51 mGy/year and 19.39 ± 7.54 mGy/year, respectively. Following dose-based stratification, cloning efficiency in non-selected (*p* = 0.16, One way ANOVA) and 6-TG selected (*p* = 0.89, One way ANOVA) T-lymphocyte cultures was comparable across the three groups, indicating similar plating efficiency and cell viability. The cloning efficiency values for each group are summarized in Supplementary Table 6.

### *HPRT* somatic mutant frequency

The mean somatic mutant frequency (MF) at the *HPRT* locus, as determined by the T-lymphocyte cloning assay was found to be comparable across the study groups. The MF (Mean ± SE, × 10^−6^) was 7.72 ± 0.87 in the NLNRA group, 8.68 ± 0.92 in the HLNRA low-dose group (LDG), and 6.96 ± 0.93 in the HLNRA high-dose group (HDG), with no statistically significant differences observed among the groups (*p* = 0.43; one-way ANOVA) (Fig. 2b.).

Of the 37 individuals, 7 (19%) were smokers (NLNRA, n = 1; HLNRA, n = 6). Alcohol consumption was common in the NLNRA group (n = 9, 75%) than in the HLNRA group (n = 13, 52%). Neither smoking (*p* = 0.523) nor alcohol use (*p* = 0.659) showed a statistically significant association with *HPRT* mutant frequency (Supplementary Table 7). Approximately 370 × 10^6^ PBMCs from 37 individuals were plated in the T-cell cloning assay, where 13 of the 37 samples yielded six *HPRT* mutant colonies each, establishing a natural upper limit benchmark for uniform downstream analysis across all samples. To ensure equal representation of mutant colonies among participants, we analysed six random mutant colonies per sample. This resulted in a final dataset of 224, 6-TG-resistant *HPRT* mutant T-lymphocyte colonies. The distribution of mutants by group was as follows: NLNRA (n = 71), HLNRA–LDG (n = 84), and HLNRA–HDG (n = 69).

### Molecular characterization of mutations in the *HPRT* locus using Multiplex PCR

The *HPRT* mutants were subjected to multiplex PCR (m-PCR) analysis using exon-specific primers spanning exons 1 through 9. The PCR products were subsequently resolved by polyacrylamide gel electrophoresis (PAGE) to assess exon presence or absence. Based on the m-PCR analysis, the mutants were categorized into five categories: 1. Total deletion (TD): Complete absence of all exons (1–9), 2. End deletion (ED): Loss of either exon 1 or exon 9, 3. Intragenic deletion (ID): Absence of regions of internal exons between exon 2 and exon 8, 4. Band shift (BS): Altered electrophoretic mobility of amplicons due to intra-exonic deletions, 5. Non-deletion (ND) : Presence of all 9 exons, indicative of structurally intact *HPRT* gene.

### Molecular spectrum and clonality analysis of the *HPRT* mutants across different groups

Multiplex PCR analysis of 224 *HPRT* mutant T-lymphocyte colonies revealed that 163 (72.8%) had all nine exons, classified as ND mutations (which may include point mutations, small indels, or other intragenic changes). The remaining 61 mutants (27.2%) were classified as deletion mutations as they failed to amplify one or more exons. The distribution of deletion types (TD, ED, ID, BS) across NLNRA and HLNRA (LDG and HDG) groups is summarized in Table 1. Among 61 mutations, 9 exhibited complete loss of all nine exons and categorized as TD. Thirteen mutants showed end deletions (ED) involving terminal regions of the *HPRT* gene; nine had deletions at exon 1 and four at exon 9. Seventeen deletions were intragenic, involving loss of exons between 2 and 8 (ID). This included seven mutants with deletions of exons 2 and 3, and the remaining ten ID mutants exhibited diverse patterns, such as single exon deletions (involving exons 2, 4, 5, or 6), multiple exon deletions (exons 3 and 4, 5 and 6, or 7 and 8), a quadruple deletion spanning exons 3 to 6, and a quintuple deletion encompassing exons 4 to 8. The remaining 22 deletion mutants were classified as band shift mutations, indicating intra-exonic deletions. Of these, thirteen showed altered bands corresponding to deletions within exons 7 or 8, four involved exon 3, two each affected exons 1 and 2, and one involved exon 5 (Supplementary Table 8).

**Table 1:** Categorization of *HPRT* deletion mutants from NLNRA and HLNRA (LDG and HDG) using multiplex PCR patterns.

Area	Mean dose (mGy/year)	No: of individuals	No: of *HPRT* mutant colonies analysed	No: of *HPRT* Non-Deletion mutants	No: of *HPRT* Deletion mutants	Distribution of different *HPRT* deletion mutants			
						TD	ED	ID	BS
NLNRA	1.23 ± 0.17	12	71	55	16	3	3	5	5
HLNRA LDG	3.20 ± 1.51	12	84	58	26	4	5	4	13
HLNRA HDG	19.39 ± 7.54	13	69	50	19	2	5	8	4
Total		**37**	**224**	**163**	**61**	**9**	**13**	**17**	**22**

Values in bold represent the total counts.

To determine whether deletion mutations represented independent events or clonal expansions, we analysed the clonality of 14 mutants from six individuals who exhibited identical deletion profiles. T-cell receptor (TCR) γ gene rearrangement patterns were evaluated to distinguish unique mutations from clonally derived ones. Mutants with distinct TCR patterns were classified as independent, while those sharing identical patterns were considered to have originated from in vivo clonal expansion of a single event, as outlined by Nicklas et al.^35,36,52^. The analysis showed that among the fourteen mutants, 8 showed unique TCR arrangements and were classified as independent, whereas six shared identical patterns, confirming their clonal origin. Altogether, 55 of the 61 deletion mutants (90.2%) represented independent mutational events, while the remaining six (9.8%) arose through clonal expansion.

**Fig. 3:**
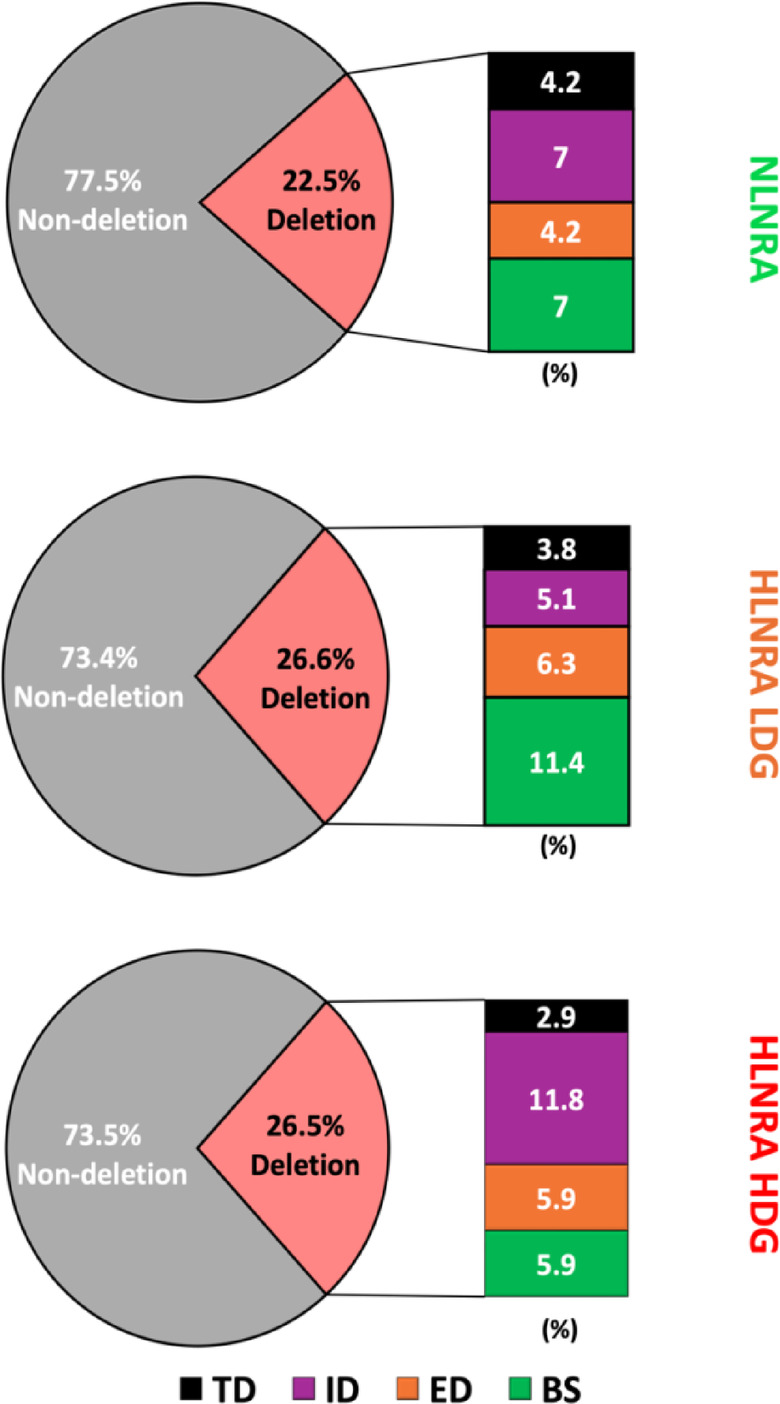
The frequencies of deletion and non-deletion *HPRT* mutants and the different types of deletion mutants in NLNRA, HLNRA LDG and HLNRA HDG.

The distribution of deletion and non-deletion mutations was compared across the three groups. In the NLNRA group, 22.5% of mutants were deletions and 77.5% were ND mutations. In the HLNRA LDG and HDG groups, deletion mutations accounted for 26.6% and 26.5%, respectively, with corresponding ND proportions of 73.4% and 73.5% (Fig. 3). These differences were not statistically significant (NLNRA vs. HLNRA: *p* = 0.52; NLNRA vs. HLNRA LDG: *p* = 0.57; NLNRA vs. HLNRA HDG: *p* = 0.59; HLNRA LDG vs. HDG: *p* = 0.99; chi-square test). Additionally, the frequencies of specific deletion types (TD, ED, ID, BS) did not differ significantly between NLNRA and HLNRA groups (TD: *p* = 0.57, ED: *p* = 0.72, ID: *p* = 0.97, BS: *p* = 0.88), between NLNRA and HLNRA LDG (TD: *p* = 0.71, ED: *p* = 0.71, ID: *p* = 0.39, BS: *p* = 0.47), between NLNRA and HLNRA HDG (TD: *p* = 0.53, ED: *p* = 0.80, ID: *p* = 0.43, BS: *p* = 0.55), or between HLNRA LDG and HDG (TD: *p* = 0.77, ED: *p* = 0.91, ID: *p* = 0.09, BS: *p* = 0.17). These findings indicate that the overall frequency and spectrum of *HPRT* deletion mutations, including their clonality status, were comparable across low and high natural background radiation exposure groups, suggesting no significant influence of chronic low-dose radiation on the structural characteristics or clonal behaviour of *HPRT* mutations.

### Mapping the extent of deletions beyond the *HPRT* locus using STS marker analysis

To further investigate the extent of deletions beyond the coding areas of the *HPRT* gene, we analysed 11 sequence-tagged site (STS) markers specific to the human Xq26 region of the X chromosome. We focused on 8 TD and 12 ED mutants, as these deletions involve the extreme ends of the *HPRT* gene, unlike in ID and BS mutants, where deletions are restricted within the *HPRT* gene. Among the 11 STS markers, five markers were located on the centromeric side of the *HPRT* gene spanning 1.75 Mbp region, and six on the telomeric side, covering 1.7 Mbp region. The PCR amplification of STS markers, followed by gel-based screening for the presence or absence of specific markers in proximity of the *HPRT* locus, allowed us to estimate the sizes of deletions in various mutants. Sizes of these deletions were determined by measuring the distance from the first and last missing PCR products.

**Fig. 4:**
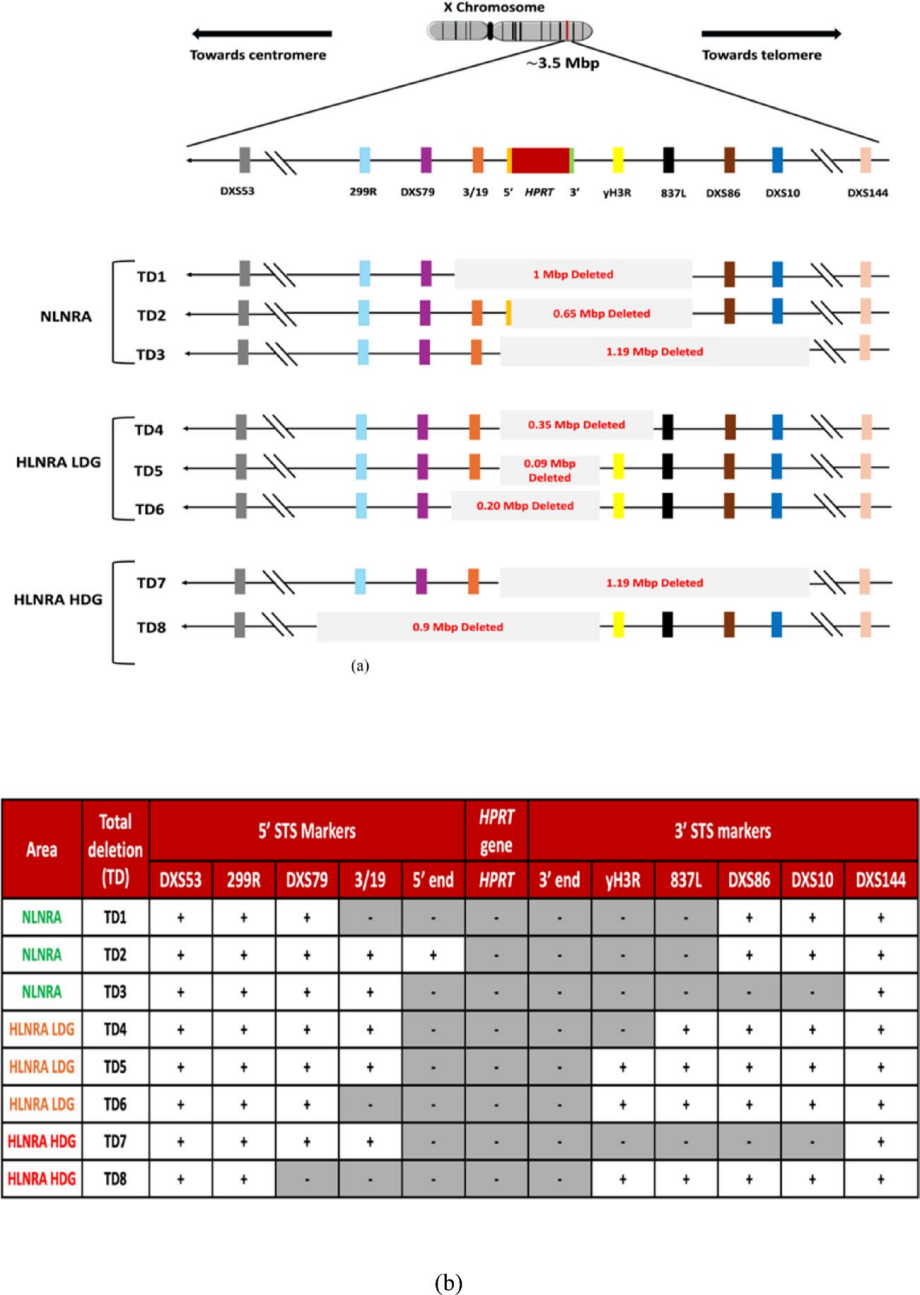
(**a**) Extent of deletion observed in Total Deletion (TD) *HPRT* mutants from NLNRA and HLNRA (LDG and HDG). (**b**): Analysis of 8 Total deletions (TD1–TD8) of *HPRT* using 11 Xq26 STS markers. The shaded areas represent the deleted segments. The presence (+) or absence (−) of individual markers is also indicated by symbols.

As shown in Figs. 4a and b, in the TD mutants, each exhibited deletion of the entire *HPRT* gene along with at least one adjacent marker, while the outermost STS markers—DXS53 (centromeric, 1.75 Mbp) and DXS144 (telomeric, 1.7 Mbp)—remained intact across all samples, thereby delineating the boundaries of deletions. In the NLNRA group, deletion sizes ranged from 0.65 to 1.19 Mbp. The largest deletion (1.19 Mbp) encompassed six markers (5′, 3′, yH3R, 837L, DXS86, and DXS10), while a 1.0 Mbp deletion spanned five markers (3/19, 5′, 3′, yH3R, and 837L). The smallest observed deletion (0.65 Mbp) included three markers (3′, yH3R, and 837L) in addition to the *HPRT*. In HLNRA (both LDG and HDG), deletion sizes among TD mutants varied from 0.09 to 1.19 Mbp. All five HLNRA TD mutants showed deletion of both 5′ and 3′ markers adjacent to the *HPRT* gene. The largest deletion (1.19 Mbp) mirrored the NLNRA case, spanning from the 5′ marker to DXS10. Another substantial deletion (0.90 Mbp) involved five STS markers extending from 299R (centromeric) to the 3′ marker. Smaller deletions included a 0.35 Mbp region removing yH3R, a 0.20 Mbp region affecting 3/19 plus the 5′ and 3′ markers, and the smallest deletion (0.09 Mbp) restricted to the 5′ and 3′ markers alone. Thus, while all eight TD mutants from both groups showed deletion of the entire *HPRT* gene and adjacent regions, the deletion size in NLNRA ranged from 0.65 to 1.19 Mbp, compared to 0.09 to 1.19 Mbp in HLNRA.

**Fig. 5:**
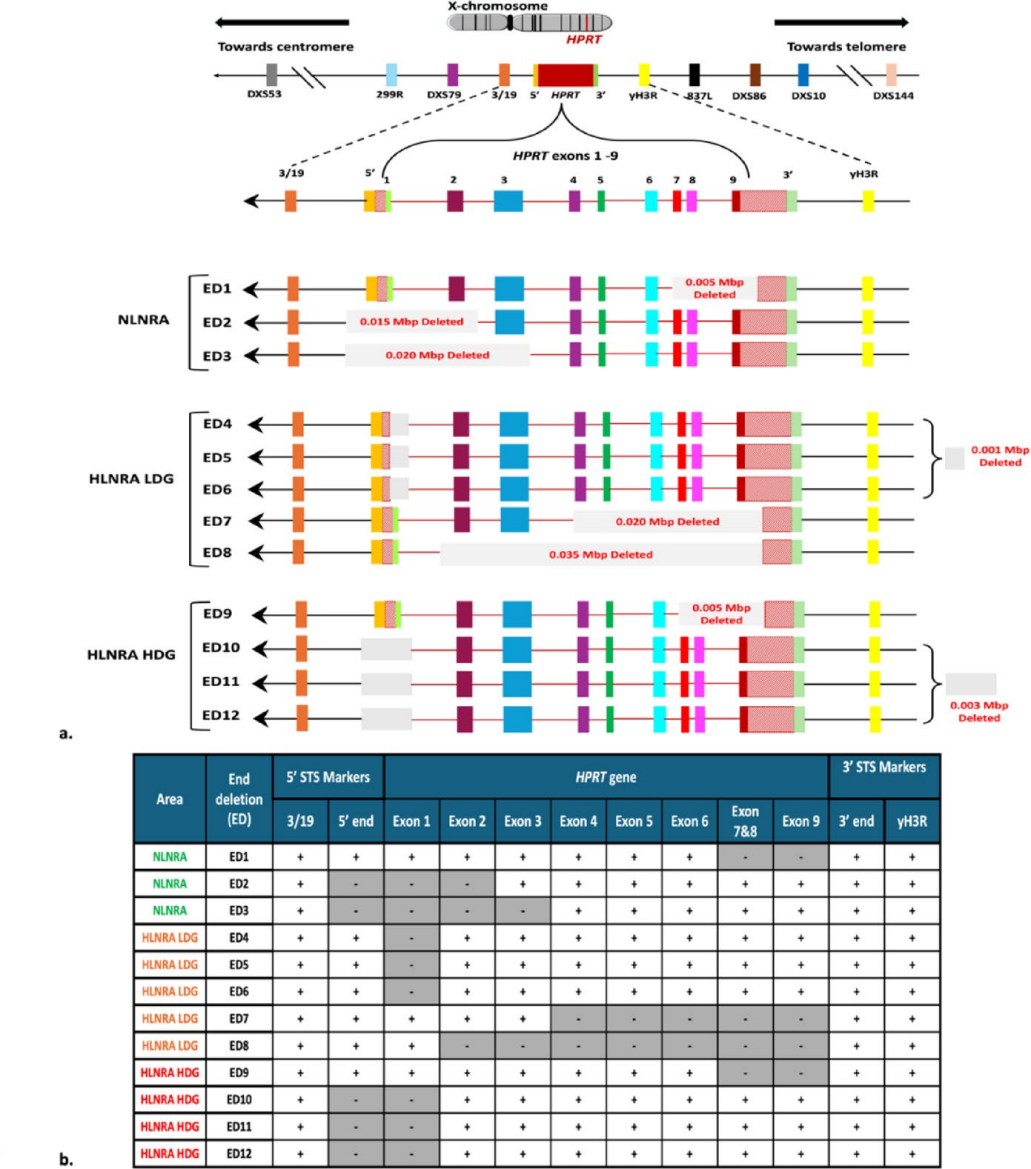
(**a**) Extent of deletion observed in End Deletion (ED) *HPRT* mutants in NLNRA and HLNRA (LDG and HDG). The deleted region is marked as an unfilled rectangle. (**b**) Analysis of 12 End deletions (ED1–ED12) of *HPRT* using 11 Xq26 STS markers. The shaded areas represent the deleted segments. The presence (+) or absence (−) of individual markers is also indicated by symbols.

In contrast, the deletion profiles of ED mutants, shown in Figs. 5a and b, revealed smaller and more localized deletions. In NLNRA, ED deletion sizes ranged from 0.005 to 0.020 Mbp, while in HLNRA they ranged from 0.001 to 0.035 Mbp. Among ED mutants involving exon 1, the adjacent 5′ STS marker was deleted in both NLNRA and HLNRA HDG, but remained intact in HLNRA LDG. Notably, ED mutants involving exon 9 uniformly retained the 3′ marker and all downstream telomeric markers across both NLNRA and HLNRA groups. These findings demonstrate a distinct deletion pattern in ED mutants, in which deletions are confined to smaller regions near gene termini, particularly affecting the 5′ end in some cases but sparing the telomeric side. Overall, these data suggest that while TD mutants display large deletions extending up to 1.19 Mbp and spanning multiple STS markers, ED mutants exhibit significantly more restricted deletions with a maximum extent of 0.035 Mbp, highlighting the differential structural impacts of these mutation types.

### Differential expression of DNA repair pathways in the *HPRT* mutants

**Fig. 6:**
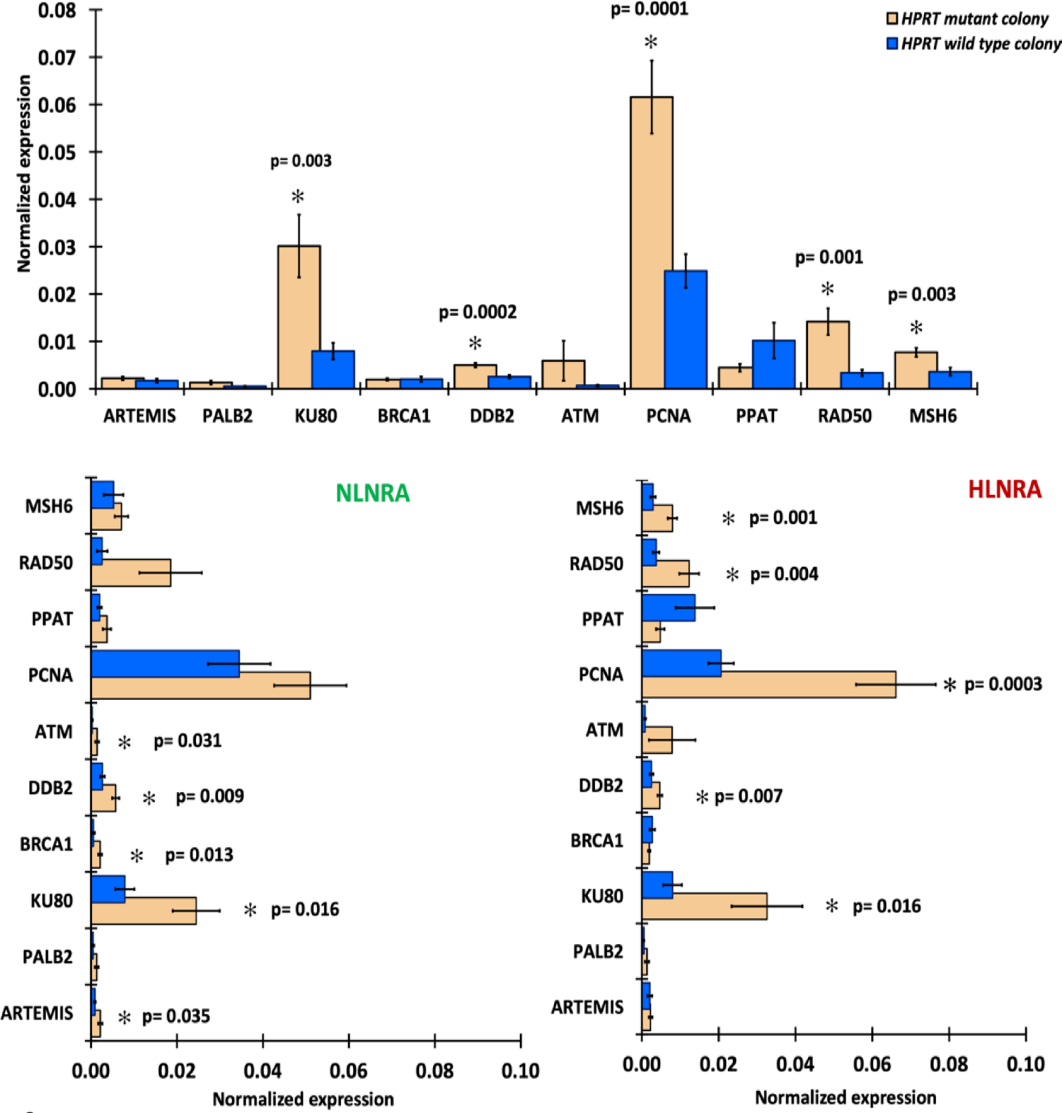
Gene expression profile of DDR and repair genes in *HPRT* mutant and wild-type lymphocyte colonies.

To evaluate whether *HPRT* mutation status is associated with altered DNA damage response (DDR) and repair gene expression, a randomly selected panel of 10 genes representing major DNA repair pathways was analyzed by RT-qPCR in *HPRT*-mutant and *HPRT* wild-type lymphocyte colonies. Overall, *HPRT*-mutant colonies exhibited significantly higher expression of several DDR and repair genes, including *KU80* (*p* = 0.003), *DDB2* (*p* = 0.0002), *PCNA* (*p* = 0.0001), *RAD50* (*p* = 0.001), and *MSH6* (*p* = 0.003), whereas *ARTEMIS, PALB2, BRCA1, ATM,* and *PPAT* showed no significant differences. Stratified analysis revealed group-specific patterns. In the NLNRA group, *HPRT*-mutant colonies showed significantly higher expression of *ARTEMIS* (*p* = 0.035), *KU80* (*p* = 0.016), *BRCA1* (*p* = 0.013), *DDB2* (*p* = 0.009), and *ATM* (*p* = 0.031) compared with wild-type colonies, while *PALB2, PCNA, RAD50, MSH6,* and *PPAT* remained unchanged. In the HLNRA group, *HPRT*-mutant colonies demonstrated significant upregulation of *KU80* (*p* = 0.016), *DDB2* (*p* = 0.007), *PCNA* (*p* = 0.0003), *RAD50* (*p* = 0.004), and MSH6 (*p* = 0.001), whereas *ARTEMIS, PALB2, BRCA1, ATM,* and *PPAT* did not differ significantly (Fig. 6.)

## Discussion

This study aimed to determine the effects of chronic low-dose ionizing radiation on a healthy population residing on the southwest coast of Kerala by examining the molecular spectrum of spontaneously occurring in vivo somatic mutations at the *HPRT* locus. The mean *HPRT* mutant frequency (MF) observed across 37 individuals from NLNRA and HLNRA (7.71 ± 0.52 × 10^−6^) was consistent with previously reported background levels in South Indian populations (8.99 ± 0.66 × 10^−6^)^53^ and aligned with findings from other studies employing the T lymphocyte cloning assay^28,31^.

In the present study, *HPRT* mutant frequency estimates obtained from a pilot subset of NLNRA (n = 5; mean MF = 9.4 × 10^−6^) and HLNRA (n = 5; mean MF = 6.08 × 10^−6^) individuals were used to estimate the theoretical sample size required to detect group differences, indicating a minimum requirement of approximately 11 individuals per group. However, upon inclusion of additional samples (NLNRA, n = 12; HLNRA, n = 25), the mean *HPRT* mutant frequencies in the two groups became comparable (NLNRA: 7.72 × 10^−6^, 95% CI 1–16; HLNRA: 7.71 × 10^−6^, 95% CI 1–19), with substantially overlapping confidence intervals, indicating that within-group variability exceeded the difference between group means.

Furthermore, the *HPRT* mutant frequency observed in the HLNRA-HDG (6.96 × 10^–6^) group was statistically comparable to that in NLNRA individuals (7.72 × 10^–6^). Notably, this result is consistent with several independent cytogenetic and molecular studies conducted in the HLNRA population of Kerala, which similarly reported no detectable increase in genomic instability associated with chronic low-dose natural radiation exposure. Collectively, the present results add to the growing body of evidence indicating that long-term exposure to high-level natural background radiation in this region does not lead to detectable increases in multiple validated biomarkers of genomic damage at the population level^13,14,18^.

Dose estimation in this study was based on external gamma radiation, as it accounts for approximately 60–70% of the total annual effective dose in Kerala’s high-level natural radiation areas^54^. Internal exposure from naturally occurring radionuclides in food, as well as from radon and thoron, remains relatively low (0.10 mSv for radon and 0.44 mSv for thoron) and exhibits limited variation across the study region^55,56^. This is largely attributable to the rapid decay of thoron (half-life 55.6 s) and low indoor radon accumulation associated with well-ventilated housing conditions. Moreover, inhalation of radon and thoron progeny predominantly results in localized radiation doses to the respiratory epithelium and is unlikely to contribute substantially to the systemic dose received by circulating peripheral blood lymphocytes. Since the T-cell cloning assay measures somatic mutations at the *HPRT* locus in circulating peripheral blood T lymphocytes and thus reflects a systemic endpoint^57^, dose estimation based on external gamma radiation provides a biologically relevant metric for assessing radiation-associated effects on lymphocyte mutant frequency in this population.

Although the overall *HPRT* mutant frequency did not differ significantly between groups, analysis of the mutation spectrum, particularly deletion events at the *HPRT* locus, provides additional insight into somatic genetic alterations potentially associated with chronic low-dose radiation exposure. Deletion mutations at the *HPRT* gene have been proposed as informative biomarkers of radiation exposure in both experimental and in vivo studies^21^. Accordingly, characterization of deletion spectra at the *HPRT* locus may help determine whether chronic low-dose radiation exposure is associated with distinct patterns of somatic genetic damage, even in the absence of changes in overall mutant frequency.

Large deletions in the *HPRT* gene, encompassing complete or multiple exons, enhance our understanding of mutagenesis mechanisms, may aid in assessing the biological impacts of environmental mutagens, and serve as a model for genomic rearrangements linked to various human conditions and diseases^58^. These substantial deletions associated with total deletion (TD), end deletions (ED), and intragenic deletions (ID) may also arise from severe genomic instability caused by exposure to ionizing radiation^59^, replication fork collapse^60^, Alu-mediated recombination^61,62^, illegitimate recombination, or chromosomal abnormalities^21^. In the present study, analysis of the deletion spectrum revealed no significant differences in the incidence of TD, ED, or ID between NLNRA and HLNRA groups. Notably, deletions involving exon 1 or exon 9 were similarly distributed across radiation dose groups. These findings align with studies from other radiation-exposed populations, such as those affected by the Chernobyl disaster, which also reported no significant increase in TD among clean-up workers (exposed individuals)^33^. The absence of increased EDs indicates that chronic low-dose exposure does not elevate the risk of terminal deletions.

In the case of intragenic deletions, exons 2 and 3 were most frequently deleted, followed by combinations such as exons 3–4, 5–6, 7–8, and multi-exon deletions (e.g., exons 3–6, 4–8). Single exon deletions were also observed for exons 2, 4, 5, and 6. The prevalence of exon 2–3 deletions have been previously linked to illegitimate activity of the V(D)J recombinase targeting cryptic signal sequences within introns 1 and 3^63^. The similar frequencies of such deletions across NLNRA and HLNRA populations further support the absence of radiation-induced clonal bias in this region. Additionally, other multiple and single-exon deletions also remained comparable among different dose groups.

Band shift (BS) mutations within the exons of the *HPRT* gene, identified by changes in PCR fragment sizes, were also comparable between the groups. Among the BS events, exons 7 and 8 were frequently affected. A plausible explanation for this observation could be that since exons 7 and 8 are co-amplified as a single fragment, the structural changes (insertions, deletions, or rearrangements) within or near these exons are more likely to result in a noticeable size shift. Altogether, the comparable rates of various deletion mutations at the *HPRT* locus across different HLNRA groups implies that chronic low-dose radiation exposure does not markedly elevate the incidence of large-scale genomic deletions.

In the analysis of the mutational spectrum at the *HPRT* locus in human lymphocytes, it is essential to ascertain that the mutations are clonally independent. This is because when a T-cell undergoes a mutation in the *HPRT* gene and is subsequently stimulated by an antigen, multiple occurrences of the same mutation are likely to be observed in the analysis of the mutational spectrum, which could lead to a potentially misleading interpretation^45^. Clonal expansions were addressed in the *HPRT* deletion mutation dataset using T cell receptor $\gamma$ gene rearrangement analysis, which ensured accurate representation of independent events.

The extent of spontaneous deletions beyond the *HPRT* locus was assessed in TD and ED mutants using 11 STS PCR primers. In line with prior studies^33,49,50–52,64–67,68–71^, our results show that TD mutations can span substantial genomic distances including the Xq26 STS markers. It has been demonstrated that large deletions of at least 0.7 Mbp are tolerated at the *HPRT* locus in in-vivo derived human T-lymphocytes^72^. In our study, six of eight TD mutants had deletions extending at least 0.35 Mb, approximately eight times the size of the *HPRT* gene. These deletions encompassed both upstream and downstream STS markers, with the most extensive spanning ~ 1.19 Mb toward the telomeric end. Nelson et al. reported, a deletion past DXS10 of about 1.2 Mb from the *HPRT* gene, involving the 342R marker have been associated with reduced cell fitness^50^. However, the mutants in our study did not exhibit long-term viability problems suggesting that the deletion is limited to the region near DXS10 and does not involve the 342R marker. Similarly, H. Wu et al. suggested that the location of the nearest essential gene towards the centromere is between 2 and 3 Mb from the *HPRT* locus^73^. The largest centromeric deletion obtained in our study has extended only up to marker 299R, located 0.75 Mb away from the *HPRT* locus, and all TDs possessed the distal marker DXS53, which is 1.75 Mb from the *HPRT* locus. Consequently, none of the TD mutants extended beyond ~ 1.75 Mb towards the centromeric region and ~ 1.19 Mb towards the telomeric region. Furthermore, the similar deletion sizes observed in NLNRA and HLNRA indicate that chronic low-dose radiation exposure does not exacerbate large-scale genomic loss at this locus. Alternatively, the misrepair or improper joining of DNA double-strand breaks (DSBs) resulting from endogenous or exogenous sources of damaging agents could contribute to the occurrence of these significant deletions^73^. However, within HLNRA, the LDG and HDG showed deletion extents of 0.09 to 0.35 Mb and 0.9 to 1.19 Mb, respectively. The difference in extent of deletions observed in the LDG of HLNRA compared to NLNRA and HDG requires further study to elucidate the contributing factors.

In EDs involving exon 1 of the *HPRT* gene in NLNRA and HDG a closely associated neighbouring marker of the *HPRT* gene (the 5’ end marker) was deleted. Since exon 1 and the 5’ end marker are in close proximity (approximately 1.67 Kbp), deletions in exon 1 are likely also to affect the neighbouring marker. However, we did not observe the 5’ adjacent marker deletions in the LDG of HLNRA, which appears to be of interest and necessitates further examination. In contrast, no STS markers were deleted in the EDs involving exon 9 of the *HPRT* gene in NLNRA and HLNRA (LDG & HDG). Furthermore, exon 9 and the 3’ end marker are separated by a considerable distance (approximately 14.57 Kbp), suggesting that deletions in exon 9 might not influence the adjacent markers.

In addition to characterizing structural deletion patterns at the *HPRT* locus, we performed transcriptional profiling using a targeted panel of 10 representative DNA damage response (DDR) and repair genes to obtain a focused view of pathway-level changes associated with *HPRT* mutation status. This analysis revealed consistent differences in gene expression in *HPRT*-mutant lymphocyte colonies, including elevated expression of *KU80, RAD50, DDB2, MSH6*, and *PCNA*, compared with wild-type colonies grown under identical culture conditions. The coordinated nature of these transcript-level changes suggests an altered DNA damage response landscape in *HPRT* mutant colonies and may also reflect an adaptive transcriptional response. Overall, these findings may indicate that the observed transcriptional changes are downstream responses related to *HPRT* mutation status. However, given the cross-sectional and correlative nature of the study, these findings do not establish a causal role for *HPRT* deficiency in regulating DNA repair pathways, nor do they permit inference of temporal relationships or functional consequences. Thus, while the observed expression patterns support an association between *HPRT* mutation status and changes in the cellular repair machinery, targeted functional validation studies (such as CRISPR knockout, protein-level analyses, or DNA repair capacity assays) will be needed to determine whether these transcriptional changes translate into altered DNA repair function.

## Limitations

This study characterizes somatic mutations and the deletion spectrum at the *HPRT* locus in individuals chronically exposed to low-dose natural radiation along the southwest coastal region of Kerala and examines their association with DNA damage response (DDR) and repair gene expression. The findings should be interpreted in light of several limitations. Although a pilot-based sample size estimate was performed, the smaller difference in mutant frequency across the full cohort limited statistical power to detect subtle effects. Dose stratification was based on external gamma radiation; internal exposure from inhalation and ingestion, while reported to be low in this region, was not quantified. Smoking and alcohol consumption data were collected (Supplementary Table 9) and incorporated into multivariable analyses; however, their uneven distribution and low prevalence constrained the ability to fully assess potential confounding. While the single-locus *HPRT* assay is a sensitive and well-established biomonitoring tool, genome-wide approaches such as whole-genome or whole-exome sequencing would provide greater sensitivity for detecting broader mutational changes. In addition, targeted sequencing of non-deletion mutants would provide more comprehensive insights on molecular mutation spectrum and will be focussed in future studies. Finally, the cross-sectional design limits causal inference, particularly with respect to temporal relationships between *HPRT* mutation status and DDR gene expression changes. Despite these limitations, this study leverages real-world human population data and provides valuable insights into somatic mutational patterns under chronic low-dose radiation exposure, highlighting the need for future studies incorporating larger cohorts, comprehensive dosimetry, genome-wide analyses, and longitudinal designs to better resolve causality and subtle biological effects.

## Conclusion

Our study demonstrates that the overall mutational spectrum and deletion profiles at the *HPRT* locus did not differ significantly between populations from NLNRA and HLNRA (LDG & HDG). The extent of deletions, including those exceeding 1 Mb, remained similar across dose groups, indicating that chronic low-dose exposure to natural background radiation does not lead to increased large-scale genomic alterations at this locus. Importantly, while this work focused on deletion mutants, approximately 75% of spontaneous *HPRT* mutants were non-deletion types. Future sequencing-based analysis of these mutants will be critical to uncovering subtle genomic changes and providing deeper mechanistic insights into the long-term biological effects of chronic low-dose radiation.

This study contributes valuable human data on populations chronically exposed to natural radiation and highlights that elevated background radiation along the Kerala coast does not significantly alter somatic mutation frequency or the molecular spectrum at the *HPRT* locus. Beyond its immediate findings, the work underscores the importance of continued research into mutagenesis and radio-adaptive mechanisms under low-dose exposure scenarios. Such knowledge is essential not only for understanding the interplay between genome stability and environmental stress but also for refining radiation protection frameworks, advancing health risk assessment, and strengthening environmental safety protocols and regulatory standards.

## Supplementary Information

Below is the link to the electronic supplementary material.

Supplementary Material 1

Supplementary Material 2

## Data Availability

Data will be available upon reasonable request to the corresponding author.
