# Supplementary material for: Molecular analysis of somatic mutations at the HPRT locus in lymphocytes of human population exposed to chronic high background natural radiation
Source: Sci Rep. 2026 Mar 16;16:13709. doi: 10.1038/s41598-026-43100-y (PMC13125333; doi:10.1038/s41598-026-43100-y)

**Supplementary Figure 1**: Physical map of the region of Xq26 surrounding the *HPRT* locus. 11 STS markers were used to determine the extent of the deletions in Total and End deletion *HPRT* mutants. The *HPRT* locus is oriented such that the 5' end is centromeric and the 3' end is telomeric. The most proximal marker used in this study, DXS53, has been mapped to $\sim$1.75 Mb upstream of *HPRT*, and the most distal marker DXS144, is mapped $\sim$1.7 Mb downstream of *HPRT*, covering a region of $\sim$3.5Mb.


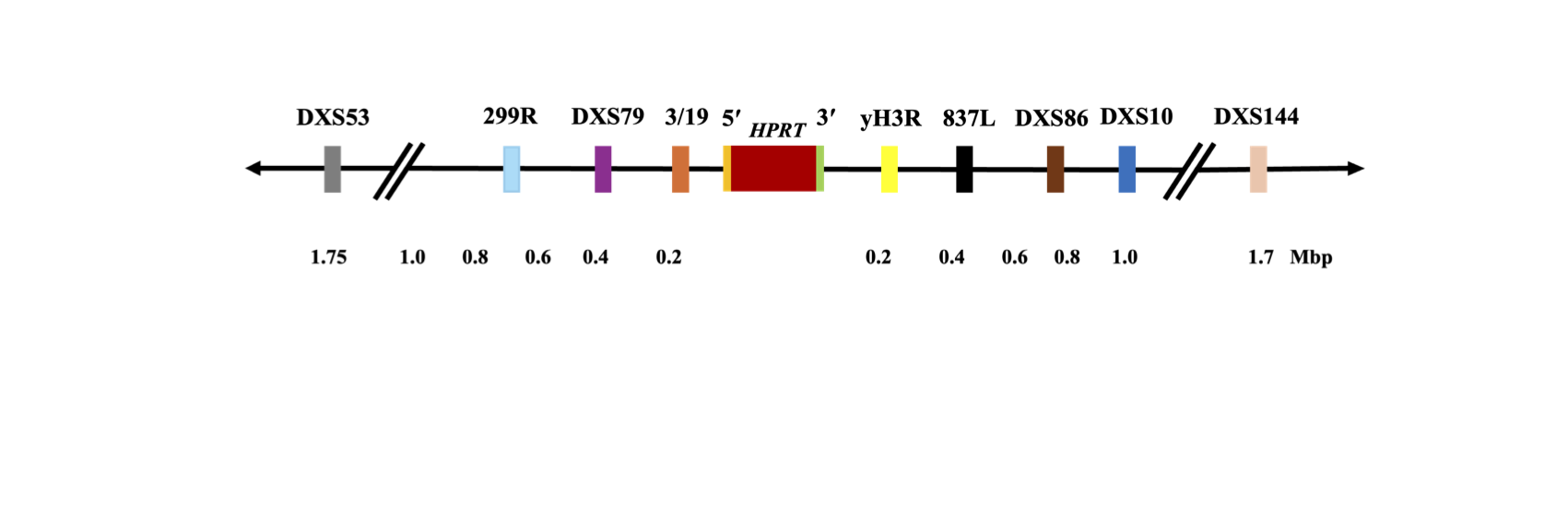


**Supplementary Figure 2**: Representative RT-qPCR amplification and melt curves showing specific amplification and product purity for target gene expression analysis.


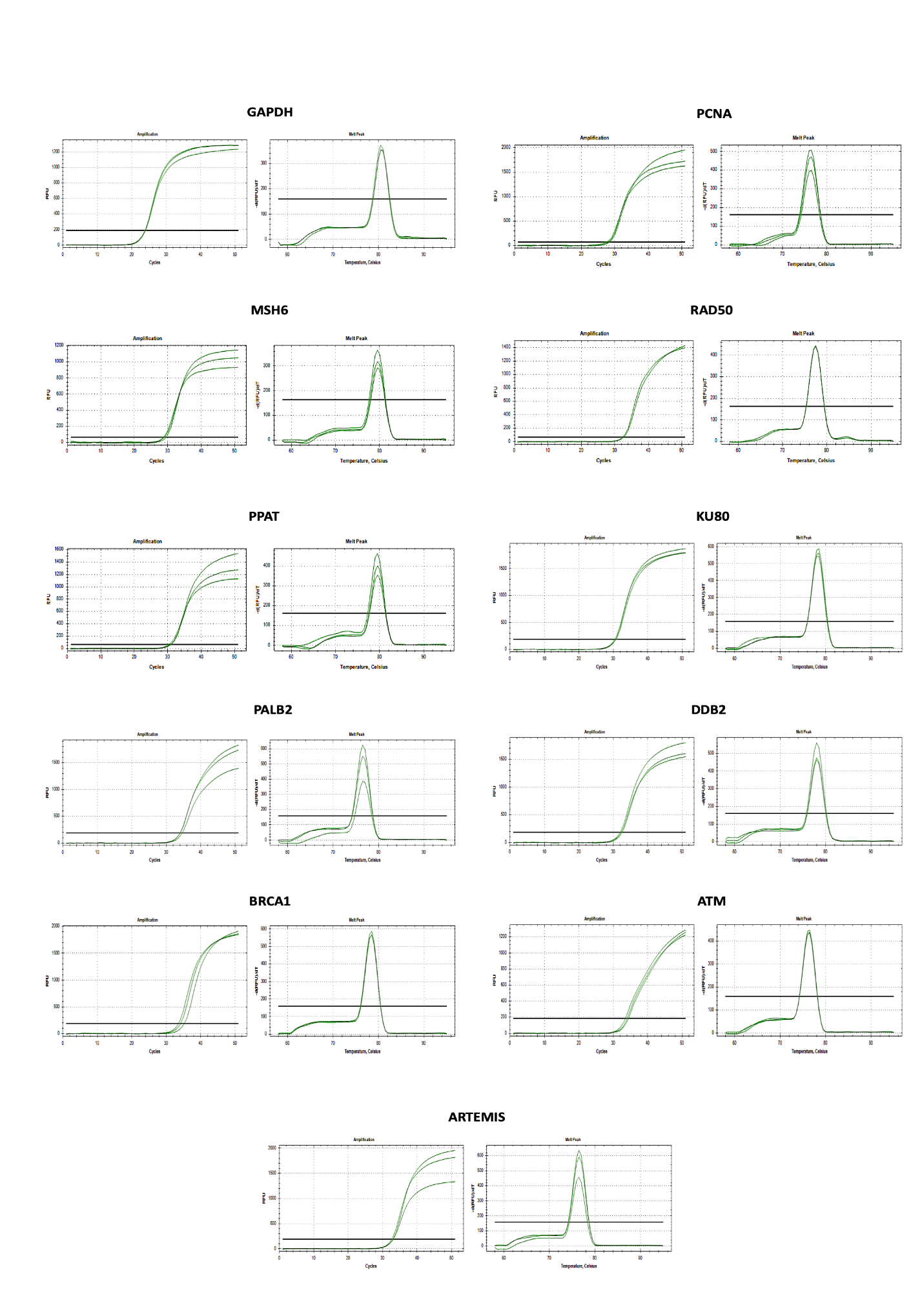

Supplement: Supplementary file 1 — Supplementary Material 1 [file 41598_2026_43100_MOESM1_ESM.docx]
