# Supplementary material for: Molecular analysis of somatic mutations at the HPRT locus in lymphocytes of human population exposed to chronic high background natural radiation
Source: Sci Rep. 2026 Mar 16;16:13709. doi: 10.1038/s41598-026-43100-y (PMC13125333; doi:10.1038/s41598-026-43100-y)
Supplement: Supplementary file 2 — Supplementary Material 2 [file 41598_2026_43100_MOESM2_ESM.docx]

**Supplementary Table 1: Primer sequences utilized for the multiplex PCR amplification of *HPRT* exons 1 to 9**

| **S.No:** | ***HPRT* exon** | **Forward primer** | **Reverse primer** | **Amplicon size (bp)** |
| --- | --- | --- | --- | --- |
| 1. | 1 | TGGGACGTCTGGTCCAAGGATTCA | CCGAACCCGGGAAACTGGCCGCCC | 626 |
| 2. | 2 | CCTGTAATGCTCTCATTGAAACA | GCTGCTGATGTTTGAAATTAACAC | 211 |
| 3. | 3 | GTTTAATGACTAAGAGGTGTTTG | GAAAACCTACTGTTGCCACTAAA | 311 |
| 4. | 4 | GTGTGTGTACATAAGGATATACA | TTCTTCCCTTTCAAGATACATAC | 166 |
| 5. | 5 | GGAAATACCGTTTTATTCATTGT | GTGCATACTAAGTTAGAAAAGTC | 127 |
| 6. | 6 | GTGACTCTGAATTTAAAGCTATG | CTGTGTCAAAATGTCATACATAC | 150 |
| 7. | 7&8 | GTCTCTCTGTATGTTATATGTCAC | TGCGTGTTTTGAAAAATGAGTGAG | 379 |
| 8. | 9 | GCTATTCTTGCCTTTCATTTCAG | CAAACTCAACTTGAACTCTCATC | 136 |

**Supplementary Table 2: Primer sequences for multiplex PCR amplification of TCR**$\boldsymbol{\gamma}$ **gene**

| **S.No:** | **Gene segment** | **Primer sequence** |
| --- | --- | --- |
| 1. | Vγ I | GAAGCTTCTAGCTTTCCTGTCTC |
| 2. | Vγ II | GAAAGGAATCTGGCATTCCG |
| 3. | Vγ III &IV | TCATTCACTGGTACCGGCAGAAACCAAA |
| 4. | Jγ1/2 | CGTCGACAACAAGTGTTGTTCCAC |
| 5. | JPγ1/2 | TTACCAGGTGAAGTTACTATGAGC |
| 6. | JPγ | ATGATAAGCTTTGTTCCGG |

**Supplementary Table 3: Sizes of PCR-amplified junctional regions of rearranged TCR** $\boldsymbol{\gamma}$ **genes, measured in base pairs (bp), involving various V and J gene segment subgroups.**

| **Subgroups in V or J gene segments** | **Jγ1/2** (50bp) | **JPγ1/2** (64bp) | **JPγ** (57bp) |
| --- | --- | --- | --- |
| **Vγ I** (462bp) | 512 | 526 | 519 |
| **Vγ II** (131bp) | 181 | 195 | 188 |
| **Vγ III &IV** (211bp) | 261 | 275 | 268 |

**Supplementary Table 4: Primer sequences used for amplifying STS markers in mapping the end regions of *HPRT* total and end deletion mutants.**

| **S.No:** | **STS Marker** | **Forward primer** | **Reverse primer** | **Amplicon size (bp)** |
| --- | --- | --- | --- | --- |
| 1. | 5ʹ end | ATTCTCGTAAAACTCTTCATGGCA | ATTCTCAGATAGGTCAGGTAAGCA | 464 |
| 2. | 3ʹ end | CAGTGCACTTCCCAACAGCCAACAG | TTGATGCTCTGTTAAGTACATAAGTG | 355 |
| 3. | DXS79 | CTGAACAGGAATAACTTGACA | AGGATTACTACAGAGGGGAT | 355 |
| 4. | 3/19 | GCCCATGAATTGAAATGAATGCC | CAGTGTTTCAGTCTTCACCAGGAC | 127 |
| 5. | 299R | TTCATTTTAGGGATGCTTGGATG | ATCAGCACAAACTAGTGTCTTCC | 297 |
| 6. | DXS53 | TTGGCACTTGGCATAATAAT | TCAAACTCCTCTTCGGGTAA | 177 |
| 7. | 837L | AAGCATTAGTTACCCCAACCTCT | AACACTCCCTTGAATGCAGAATG | 405 |
| 8. | yh3R | AGCATAGAGCTTATTACTGGATTG | CCAACCATCAACCTGGCCTGACTC | 130 |
| 9. | DXS86 | TGACACCATTTACCTAATGCAC | GTTGCAGAGGCACGATTGGAACT | 694 |
| 10. | DXS10 | ACCTTGATCTTTCACCTATGGCTG | CACATTCAGACTTCCTCAGGGACA | 438 |
| 11. | DSX144 | ACT GGG GCC TGA AAT GCT T | TGA TCC TCC TGC CAC AAT G | 263 |

**Supplementary Table 5: Primers used in RT-qPCR**

| **S.No:** | **Gene** | **Forward primer** | **Reverse primer** |
| --- | --- | --- | --- |
| 1. | ARTEMIS | GAGCTAGAACAGTTCACCGAGAC | CAGGCTGCTTTTCTGATACTGCA |
| 2. | ATM | TGTTCCAGGACACGAAGGGAGA | CAGGGTTCTCAGCACTATGGGA |
| 3. | BRCA1 | CTGAAGACTGCTCAGGGCTATC | AGGGTAGCTGTTAGAAGGCTGG |
| 4. | DDB2 | CTCCTCAATGGAGGGAACAA | GTGACCACCATTCGGCTACT |
| 5. | KU80 | GTTCTAAAGGTCTTTGCAGCAAGA | AAAAGCCACGCCGACTTGAGGA |
| 6. | PALB2 | GGAGCTGCATAAACATTCCGTCG | CTACGGAACAGGAACCTGAAGG |
| 7. | GAPDH | GTCTCCTCTGACTTCAACAGCG | ACCACCCTGTTGCTGTAGCCAA |
| 8. | PCNA | CAAGTAATGTCGATAAAGAGGAGG | GTGTCACCGTTGAAGAGAGTGG |
| 9 | PPAT | GCGATTGAAGCACCTGTGGATG | CGGTTTTTACACAGCACCTCCAC |
| 10. | RAD50 | GGAAGAGCAGTTGTCCAGTTACG | GAGTAAACTGCTGTGGCTCCAG |
| 11. | MSH6 | AAGGACTGGCAGTCTGCTGTAG | CGGCAACAGAATTACTGGGCGA |

**Supplementary Table 6: Cloning efficiency in non-selected and selected T-lymphocyte cultures across study groups**

| **Dose Group** | **Non-selected CE** | **6-TG Selected CE** |
| --- | --- | --- |
| **NLNRA** | 0.25±0.02 (range: 0.14-0.35) | 1.86±0.23 (range: 0.73-3.23) |
| **HLNRA LDG** | 0.20±0.03 (range: 0.07-0.34) | 1.70±0.29 (range: 0.52-3.12) |
| **HLNRA HDG** | 0.26±0.02 (range: 0.18-0.37) | 1.86±0.29 (range: 0.31-3.9) |

**Supplementary Table 7: Regression analysis of selected covariates**

| **Variable** | **Regression coefficient** | **SE** | ***t*** | **P Value** |
| --- | --- | --- | --- | --- |
|  |  |  |  |  |
| **Residential area (NLNRA/HLNRA)** | -0.002 | 1.192 | -0.002 | 0.999 |
| **ALCOHOLISM** | -0.508 | 1.140 | -0.445 | 0.659 |
| **SMOKING** | -0.948 | 1.467 | -0.646 | 0.523 |

| **Dose**  **Group** | ***HPRT* mutation spectrum** | | | | | | | | | | | | | | **Total No: of mutants** |
| --- | --- | --- | --- | --- | --- | --- | --- | --- | --- | --- | --- | --- | --- | --- | --- |
|  | **Deletion** | | | | | | | | | | | | | **Non-deletion** |  |
|  | **TD** | **ED** | | **ID** | | **BS** | | | | | | | |  |  |
|  |  |  |  |  |  | ***HPRT* exons (1-9)** | | | | | | | |  |  |
|  |  | **5'** | **3'** | **Single exon** | **Multiple exon** | **1** | **2** | **3** | **4** | **5** | **6** | **7/8** | **9** |  |  |
| **NLNRA** | 3 | 2 | 1 | 3 | 2 | - | 1 | 1 | - | - | - | 3 | - | 55 | **71** |
| **HLNRA LDG** | 4 | 3 | 2 | 1 | 3 | - | 1 | 2 | - | 1 | - | 9 | - | 58 | **84** |
| **HLNRA HDG** | 2 | 4 | 1 | 1 | 7 | 2 | - | 1 | - | - | - | 1 | - | 50 | **69** |
| **Total** (%) | **9**(4) | **9**(4) | **4**(1.8) | **5**(2) | **12**(5) | **2**(1) | **2**(1) | **4**(1.8) | **-** | **1**(0.4) | **-** | **13**(6) | **-** | **163**(73) | **224** |

**Supplementary Table 8: Spectrum of *HPRT* mutations observed across different dose groups**

**TD-**Total Deletion**, ED-**End Deletion**, ID-**Intragenic Deletion**, BS-**Bandshift

**Supplementary Table 9: Lifestyle and occupational data by dose group**

| Dose Group | Sample Code | Smoking  (Y/N) | Alcohol use  (Y/N) | Occupation |
| --- | --- | --- | --- | --- |
| NLNRA | H1 | N | Y | GE |
| NLNRA | H2 | N | N | GE |
| NLNRA | H3 | N | Y | GE |
| NLNRA | H4 | N | Y | FM |
| NLNRA | H5 | N | Y | FM |
| NLNRA | H6 | N | Y | FM |
| NLNRA | H7 | N | N | CW |
| NLNRA | H8 | N | Y | CW |
| NLNRA | H9 | N | Y | CW |
| NLNRA | H10 | N | N | CW |
| NLNRA | H11 | N | Y | FM |
| NLNRA | H12 | Y | Y | CW |
| HLNRA LDG | H13 | N | N | CW |
| HLNRA LDG | H14 | N | N | CW |
| HLNRA LDG | H15 | Y | Y | CW |
| HLNRA LDG | H16 | N | Y | FM |
| HLNRA LDG | H17 | N | N | CW |
| HLNRA LDG | H18 | N | Y | BS |
| HLNRA LDG | H19 | N | N | CW |
| HLNRA LDG | H20 | N | N | FM |
| HLNRA LDG | H21 | Y | N | CW |
| HLNRA LDG | H22 | N | N | GE |
| HLNRA LDG | H23 | N | Y | FM |
| HLNRA LDG | H24 | N | Y | CW |
| HLNRA HDG | H25 | N | Y | GE |
| HLNRA HDG | H26 | Y | Y | CW |
| HLNRA HDG | H27 | N | Y | CW |
| HLNRA HDG | H28 | N | N | CW |
| HLNRA HDG | H29 | N | Y | FM |
| HLNRA HDG | H30 | N | N | BS |
| HLNRA HDG | H31 | Y | N | BS |
| HLNRA HDG | H32 | N | Y | FM |
| HLNRA HDG | H33 | N | N | CW |
| HLNRA HDG | H34 | N | N | FM |
| HLNRA HDG | H35 | Y | Y | FM |
| HLNRA HDG | H36 | N | Y | FM |
| HLNRA HDG | H37 | Y | Y | CW |

Occupation: **FM**-Fishermen, **CW**-Casual worker, **GE**-Government Employee, **BS**-Business
